# Embodied cognition and STEM learning: overview of a topical collection in CR:PI

**DOI:** 10.1186/s41235-017-0071-6

**Published:** 2017-09-12

**Authors:** Steven M. Weisberg, Nora S. Newcombe

**Affiliations:** 1 Center for Cognitive Neuroscience, University of Pennsylvania, Philadelphia, PA 19104 USA; 2 Department of Psychology, Temple University, Philadelphia, PA 19122 USA

**Keywords:** Embodied cognition, STEM learning, Gesture, Education, Analogy, Action

## Abstract

Embodied learning approaches emphasize the use of action to support pedagogical goals. A specific version of embodied learning posits an action-to-abstraction transition supported by gesture, sketching, and analogical mapping. These tools seem to have special promise for bolstering learning in science, technology, engineering, and mathematics (STEM) disciplines, but existing efforts need further theoretical and empirical development. The topical collection in *Cognitive Research: Principles* includes articles aiming to formalize and test the effectiveness of embodied learning in STEM. The collection provides guideposts, staking out the terrain that should be surveyed before larger-scale efforts are undertaken. This introduction provides a broader context concerning mechanisms that can support embodied learning and make it especially well suited to the STEM disciplines.

Recent cognitive theory, under the umbrella term *embodied cognition*, has emphasized the role that the body and environment play in cognitive processing (e.g., Barsalou, 1999; 2008; Clark, 1999; 2001; Shapiro, 2011). While there are several “flavors” of embodied cognitive theory, all challenge the conception of human cognition as amodal and abstract, uncoupled from the concrete world. Considering the role of the body in human cognitive function has led to basic insights in cognitive science regarding the role of embodied tools: gesture, action, and analogical mapping. These embodied tools could be leveraged to improve learning in several ways. An embodied framework for cognition provides an opportunity for science, technology, education, and mathematics (STEM) disciplines to include embodied learning tools to enhance pedagogy. STEM education initiatives may particularly benefit from incorporating embodied cognitive principles because STEM disciplines rely on representation systems that require sensory encoding (e.g., visualizations of data and information including maps, blueprints, graphs, charts), and are nevertheless dependent on highly abstract, formalized symbol systems (e.g., those used in math or chemistry). Students need a “way in” to linking sensory representations with abstractions.

The purpose of this topical collection is to bring together theoretical discussions of how embodiment can inform educational practices in the STEM disciplines with empirical examinations of whether or not such practices actually work. While much research remains to be done, we hope that this collection provides a theoretical and empirical framework on which a more embodied pedagogy can be built. In this overview, we begin with a brief primer on what we mean (and do not mean) by embodied cognitive theory, and then develop several themes that we see in this literature.

## A brief primer on embodied cognition

J.J. Gibson (1979) speculated that, because perception is for action, cognitive science does not need a theory of representation. Perception instead could be understood in terms of sensory information—visual, kinesthetic, olfactory, et cetera. Many theories have arisen from Gibson’s theoretical starting point (i.e., that cognition may consist of nonsymbolic, sensorimotor processes as well as or even instead of symbolic representations). Generally, in the embodied view, the cognitive processes that distill and then operate on representations cannot be isolated from the sensory systems that create the representations. Arising from this general definition, however, are several flavors of embodied cognition (e.g., Clark, 1999, 2001; Shapiro, 2011; Varela, Thompson, & Rosch, 1991; Wilson, 2002).

Theorists have distinguished between embodied cognition as incorporating the role of the body in providing sensory inputs to the cognitive system—a definition not incompatible with the computational account of cognition—and embodied cognition in which the body provides a constitutive component of cognition (c.f. Kiverstein, 2012; Marshall, 2014). Clark (1999) espouses a view of cognition which incorporates body-based sensorimotor information as constitutive of cognition, taking place outside the brain. Others support a radical embodied cognitive science account that is antirepresentationalist (e.g., Chemero, 2009; Gibson, 1979; but see Clark, 1999; Fodor, 1983). These latter definitions of embodied cognition challenge the foundations of the computational approach to cognition. For a deeper review of the differences between embodied theories, as well as what constitutes strong and weak embodiment, see DeSutter and Stieff (2017)^1^ and Abrahamson and Bakker (2016)^2^.

Here, we adhere to the definition of embodied cognition as emphasizing the body’s role in forming cognitive representations. Construing cognition in this context portrays learners as poised to incorporate sensorimotor information. Their cognitive systems are affected, even constrained, by action and perception in ways traditional cognitive theorists had not considered. Embodied cognitive approaches to learning, therefore, predict that sensorimotor processes, including perception and action, should strengthen learning when included in a structured lesson, given their close and unique relationship to the cognitive system. How might this strengthening occur? We organize the remainder of this introduction in terms of possible mechanisms: 1) building analogies between sensorimotor and abstract domains; 2) using gesture as a linking and abstraction tool; 3) improving cognitive skills and abilities; 4) off-loading cognitive processes and representations into the body or environment; and 5) constructing and interpreting visual representations.

## Building embodied analogies

Analogies aid learning by mapping an unfamiliar domain onto a familiar one to encourage inferences or reach new conclusions (Gentner, 1983). Analogies do not require embodied learning tools, but embodied learning tools follow the principle of analogical learning by mapping a familiar domain onto an unfamiliar one; by moving action to abstraction (Goldin-Meadow & Beilock, 2010). Embodied learning could allow learners to extend body-based representations, familiar and easily understandable in sensorimotor terms, to map onto more abstract (or less embodied) concepts. Action and gesture can both be used to promote analogical reasoning, but a subset of verbal analogies and metaphors can specifically capitalize on embodied concepts (Lakoff & Johnson, 1980).

The symbol grounding problem, as described by Glenberg (1997) and Barsalou (1999), states that abstract representations cannot be represented abstractly ad infinitum but must ultimately be represented in a grounded form that derives from the sensorimotor system. Grounding abstract symbols in sensory or body-based representations provides the learner a way to put information in a format that can be understood and used. Using analogies to support learning in the STEM disciplines has been highly developed in mathematics; in particular, see Tran, Smith, and Buschkuehl (2017)^3^.

Nathan and Walkington (2017)^4^ propose a theory which they term a grounded and embodied theory of mathematical cognition (GEMC). They focus on the transduction of sensorimotor actions into cognitive states. In this way, concrete sensory representations can build analogies, which in turn support thinking about abstract mathematical proofs. In their theory and pilot study, Nathan and Walkington provide a demonstration that the specific information content in student gestures and directed actions supports meaningful insights—gestures are more than hand-waving—and must connect underlying concepts, pedagogical language, and student understanding.

Dove (2011) and Chatterjee (2010) have both offered continuum-based arguments which suggest that embodied, sensory representations provide concrete, perceptually grounded information, while more abstract, conceptual representations trade up in increased flexibility. In any case, embodied cognitive approaches to learning that capitalize on grounding abstract symbols in sensory modalities could be enhancing learning through the creation of an analogy. That is, an unfamiliar domain—abstract and not related directly to a sensory modality—is mapped onto a familiar domain—concrete, able to be directly perceived (c.f., Jamrozik, McQuire, Cardillo, & Chatterjee, 2016).

Applying these ideas to STEM learning, Hayes and Kraemer (2017)^5^ link theories from cognitive neuroscience about semantic knowledge and body-based sensory representations, to propose that neural signatures can be queried to examine STEM learning. In their review, Hayes and Kraemer (2017) draw on theories of neural processing, including Hebbian learning and predictive coding, to propose that sensorimotor contingencies are mapped more directly onto neural targets, while abstractions are more flexible but more difficult to acquire and more fragile. The links between STEM learning and the role of embodied analogies are still being explored, but provide an important framework for future work in cognitive neuroscience.

## The role of gesture

One way analogies can connect abstract concepts with concrete sensory representations is through gesture. Gestures are nonverbal representational movements, usually of the hands, and usually accompanying speech. Gesture plays a role in maintaining or recalling visual imagery, simulating action, and representing the speaker’s nonverbal thoughts (for a review, see Hostetter & Alibali, 2008). As Alibali and Nathan (2011) have suggested, gestures may be a way to ground abstract instructional information presented by a teacher in the physical world. Gesturing has been extensively studied as both the mechanism through which embodied concepts can be communicated, and as a way in which spatial, abstract, or physical information is encoded (Tversky, 2009). By linking linguistic and sensorimotor representations, gesture could be a powerful tool in augmenting STEM learning. Atit, Weisberg, Newcombe, and Shipley (2016)^6^ demonstrate the nuanced relationship between gesture and language in the context of learning topographic maps, an important and complex representational format for the geosciences.

Gestures are inherently spatial, and thus might help build strong spatial analogies to nonspatial domains. Cooperrider, Gentner, and Goldin-Meadow (2016)^7^ studied spontaneous student gestures during explanations of abstract concepts. Students were exposed to and instructed in causal systems (like positive and negative feedback loops) across domains, after which they were instructed to explain the differences between them. Intriguingly, and despite not being instructed to gesture, students were highly likely to describe abstract causal concepts in terms of spatial language and with spatial gestures (even when all spatial language was stripped from the instruction). These findings reveal the strength of spatial analogies for abstract concepts, and promote the importance of gesture for highlighting and emphasizing analogies.

## Improving cognitive skills and abilities

Embodied learning has the potential to improve learning generally by supporting and improving the skills of the learner. Incorporating the framework of embodied cognition into STEM learning may also improve learning by bolstering cognitive skills, or providing additional or alternative strategies. By analyzing embodied representations—through actions or gestures—educators can more effectively measure student strategy choice.

One of the principle ways that embodied tools may enhance STEM learning is by improving spatial cognition (Clifton, Chang …Mazalek, 2016; DeSutter and Stieff 2017)^1,^ 8. Spatial cognition encompasses the set of cognitive processes involved in reasoning about spatial problems. Spatial cognition has been strongly linked to entrance into STEM disciplines, and is also correlated with performance in those disciplines (Wai, Lubinski, & Benbow, 2009). Many STEM disciplines, including math (Battista, 1990), physics (Pallrand & Seeber, 1984), chemistry (Ping, Decatur, Larson, Zinchenko, & Goldin-Meadow, 2012), engineering (Hsi, Linn, & Bell, 1997), and the geosciences (Ishikawa & Kastens, 2005) have been shown to incorporate large amounts of spatial reasoning skills (for an overview see National Research Council, 2006).

Importantly, spatial skills have been shown to be malleable across age groups, genders, and socioeconomic status (Uttal et al., 2013). Embodied approaches to improving spatial ability have gained traction in adults (Burte, Gardony, Hutton, & Taylor, 2017; Chu & Kita, 2011)^9^. Among toddlers and infants, there is growing evidence that active exploration of objects promotes the development of mental rotation (Möhring & Frick, 2013). In that study, toddlers show decreased looking time to the same object rotated through any angle, but look longer at a mirror-image object (indicating surprise that is not the same object). Experimentation has also demonstrated that providing motoric experience with objects for infants at 14 months can improve their mental rotation (Frick & Wang, 2014).

On the basis of these findings, Burte et al. (2017)^9^ report on a large-scale effort to import spatial skill training. They used embodied tasks in elementary schools and examined benefits to math learning. The spatial training, Think3D!, uses paper folding and origami tasks to emphasize and improve spatial thinking. After going through the intervention, elementary school students improved on specifically targeted math problems, which required visualization and which were in a real-world context. However, in this research, unlike in cases where embodied learning builds analogies, the improvement did not generalize to abstract concepts or problems.

Embedded instruction may make it easier to engage learners and shift their attention. Unlike embodied learning, embedded instruction places decontextualized information into a meaningful situation. In the geosciences, Jaeger, Wiley, and Moher (2016)^10^ devised an embedded intervention to teach earthquakes to elementary school students. In the embedded condition, students experienced a simulated earthquake during learning—rumbling sounds were played, and computers became seismographs, which had to be read during simulated seismic activity. In a control condition, learning content and timing were the same, but maps of the earthquakes were studied, and no earthquake simulation was produced. Results revealed a significant interaction—students in the embedded condition learned more from pre- to post-test than students in the nonembedded condition. Creating a rich sensory experience to embed the learner in what can otherwise be a meaningless and abstract context can serve to deepen engagement, focus attention, and generate excitement (c.f., Johnson-Glenberg & Romanowicz, 2017)^11^.

## Offloading cognition

Offloading refers to allowing the learner to store information without relying on the taxing mental resources involved in simulating movement, visual information, or anything that can be represented as such (e.g., conceptual and spatial information). By offloading, the learner may be able to use the extra cognitive resources to focus on problem solving, making inferences, or explaining to others. Embodied actions have long been thought to offload cognitive process onto the world or the body. Margaret Wilson’s (2002) synthesis of embodied theory identifies six tenets of embodied cognition and outlines the different research support for each, three of which emphasize offloading. First, Wilson states, cognition is for action. That is, systems that were once thought of as solely the domain of the brain (memory, attention, perception) evolved for an organism that perceives and acts in a three-dimensional world. Second, human beings offload cognitive work onto the environment. Human behavior often requires cognitive resources to address situations that are informationally rich. Building from the argument that cognition arises through the interaction of mind, body, and world, Wilson’s second tenet holds that manipulating the environment is a form of cognition. For instance, in trying to solve an algebra equation, a student might move like terms to one side of the equation by performing that step and writing the resulting equation. This offloads the mental (visuospatial, in this case) process from the brain onto the environment, and thus is a way of offloading cognition. Wilson’s third view is that offline cognition is body-based. When thinking about concepts or ideas that are not available, the sensory system simulates the relevant constructs in the same way as if they were present.

How might action help offload information? Kirsh and Maglio (1994) have distinguished between certain types of offloading. They introduce epistemic action in which an actor manipulates the environment to discover new information. Epistemic action is distinct from pragmatic action, in which an actor produces an action directly toward accomplishing a goal. To test whether people use epistemic actions, in which action offloads a mental process such as rotation, Kirsh and Maglio (1994) conducted research on the video game Tetris. They found that participants performed nonessential rotations and translations on Tetris pieces instead of rotating them mentally. Because they were nonessential to the goal of placing the piece in the next row, the authors argued that rotating the pieces allowed the participants to view new possible piece combinations that were not evident in other orientations. They interpreted this result as a sign that human beings naturally modify their environment to offload cognition into the world as a means of saving valuable cognitive resources.

Observational research in the geosciences has demonstrated that offloading cognition plays a role in problem solving among geology students (Kastens, Liben, & Agrawal, 2008). When students solved complex visual problems, requiring the integration of multiple viewpoints, the students were observed to rotate candidate solutions, juxtapose two potential solutions in space, and perform other actions which suggested they were using their physical environment as a way to arrange their cognitive procedures.

Although visual perception was Gibson’s (1979) focus, it typically is dismissed as fostering embodied forms of representation. In the sense of offloading cognition into the learner’s environment, however, using visual representations that map onto natural cognitive capacities should ease student difficulty. For example, algebraic notation requires replacing visual symbols with mathematical concepts. Meaning is conveyed through some perceptual features, (e.g., an above/below spatial configuration denotes division: $\frac{24}{8}$), but not others (e.g., proximity does not change the meaning of an equation: 3 + 8 = 3 + 8). Marghetis, Landy, and Goldstone (2016)^12^ devised a study to test whether participants who are familiar with algebraic notation perceive terms as solitary units. Participants’ knowledge of order of operations was assessed (3 * 8 + 4 * 2 does not equal 3 + 8 * 4 + 2), and then participants were asked to determine whether a number changed colors either within one term (i.e., across multiplication) or across terms (e.g., across addition). Results showed that participants who knew the order of operations performed the color change more quickly when it occurred within the same term. Additionally, participants’ speed of detection correlated with their accuracy on subsequent algebra tasks. The authors frame these results as revealing that algebraic knowledge manifests as perceptual training. Although it remains to be tested experimentally, teaching students to offload the difficult mental processes of commutative and associative properties into perceptual problems could support math learning more broadly.

## Visual representations

Visual presentations of data are important for all forms of scientific communication. Designing effective graphs and knowing how to interpret them is critical. Research on effective visualization of data and visual communication of STEM concepts has capitalized on Gibsonian ideas of perception for action. Michal and Franconeri (2017)^13^ show that the cognitive processes involved in graph interpretation manifest not in high-level cognition, but in knowing where to look. In an eye tracking study, participants looked at bar graphs and were asked to attend either to the size of the bar or the luminance. In single-dimension trials, the other dimension was the same for both bars. On these trials, most participants preferred to look first at one bar, their anchor point—light/dark or short/tall. In orthogonal trials, both dimensions varied, but participants were only asked about one dimension. Even when both dimensions changed, individual participants kept their preferred anchor points. This detailed analysis of what the authors call the visual routine of interpreting graphs reveals the inextricable role that visual perception plays in driving and supporting cognition.

## Conclusion

Embodied cognitive tools provide a unique opportunity to augment STEM education by adding approaches that the cognitive system can readily incorporate and internalize. STEM disciplines are well suited to the inclusion of gesture, action, and body-based metaphors due to their reliance on arbitrary or abstract symbol systems, and their study of complex, dynamic phenomena. Recent interventions have demonstrated promising success, showing efficacy of action in the realm of physics education, and gesture in mathematics and the geosciences. Future research should delineate limitations of these approaches, and determine the role of embodied learning in technology (e.g., computer science), and engineering, understudied disciplines in interdisciplinary cognitive science.

## Footnotes

1 DeSutter & Stieff (2017) is part of the topical collection on Embodied Cognition and STEM Learning.

2 Abrahamson & Bakker (2016) is part of the topical collection on Embodied Cognition and STEM Learning.

3 Tran, Smith, & Buschkuehl (2017) is part of the topical collection on Embodied Cognition and STEM Learning.

4 Nathan & Walkington (2017) is part of the topical collection on Embodied Cognition and STEM Learning.

5 Hayes & Kraemer (2017) is part of the topical collection on Embodied Cognition and STEM Learning.

6 Atit, Weisberg, Newcombe, & Shipley (2016) is part of the topical collection on Embodied Cognition and STEM Learning.

7 Cooperrider, Gentner, & Goldin-Meadow (2016) is part of the topical collection on Embodied Cognition and STEM Learning.

8 Clifton et al. (2016) is part of the topical collection on Embodied Cognition and STEM Learning.

9 Burte, Gardony, Hutton, & Taylor (2017) is part of the topical collection on Embodied Cognition and STEM Learning.

10 Jaeger, Wiley, & Moher (2016) is part of the topical collection on Embodied Cognition and STEM Learning.

11 Johnson-Glenberg & Romanowicz (2017) is part of the topical collection on Embodied Cognition and STEM Learning.

12 Marghetic, Landy, & Goldstone (2016) is part of the topical collection on Embodied Cognition and STEM Learning.

13 Michal & Franconeri (2017) is part of the topical collection on Embodied Cognition and STEM Learning.
